# Responsiveness of different MET tumour alterations to type I and type II MET inhibitors

**DOI:** 10.1002/ctm2.70338

**Published:** 2025-05-29

**Authors:** Yonina R. Murciano‐Goroff, Valentina Foglizzo, Jason Chang, Natasha Rekhtman, Ann Elizabeth Sisk, Jamie Gibson, Lia Judka, Kristen Clemens, Paola Roa, Shaza Sayed Ahmed, Nicole V. Bremer, Courtney Lynn Binaco, Sherifah Kemigisha Muzungu, Estelamari Rodriguez, Madeline Merrill, Erica Sgroe, Matteo Repetto, Zsofia K. Stadler, Michael F. Berger, Helena A. Yu, Eneda Toska, Srinivasaraghavan Kannan, Chandra S. Verma, Alexander Drilon, Emiliano Cocco

**Affiliations:** ^1^ Department of Medicine Memorial Sloan Kettering Cancer Center New York New York USA; ^2^ Department of Biochemistry and Molecular Biology Miller School of Medicine, University of Miami Miami Florida USA; ^3^ Sylvester Comprehensive Cancer Center Miami Florida USA; ^4^ Department of Pathology Memorial Sloan Kettering Cancer Center New York New York USA; ^5^ Center for Molecular Oncology Sloan Kettering Institute New York New York USA; ^6^ Clinical Computational Diagnostics Service Memorial Sloan Kettering Cancer Center New York New York USA; ^7^ Department of Oncology Sidney Kimmel Comprehensive Cancer Center Baltimore Maryland USA; ^8^ Department of Biochemistry and Molecular Biology Johns Hopkins School of Public Health Baltimore Maryland USA; ^9^ Bioinformatics Institute (BII), Agency for Science, Technology and Research (A*STAR) Singapore Singapore; ^10^ School of Biological Sciences Nanyang Technological University Singapore Singapore; ^11^ Department of Biological Sciences National University of Singapore Singapore Singapore

**Keywords:** MET, resistance to targeted therapy, type I and type II TKIs

## Abstract

**Background:**

Mutations in c‐MET receptor tyrosine kinase (MET) can be primary oncogenic drivers of multiple tumour types or can be acquired as mechanisms of resistance to therapy. MET tyrosine kinase inhibitors (TKIs) are classified as type I or type II inhibitors, with the former binding to the DFG‐in, active conformation of MET, and the latter to the DFG‐out, inactive conformation of MET. Understanding how the different classes of MET TKIs impact tumours with varied MET alterations is critical to optimising treatment for patients with MET altered cancers. Here, we characterise MET mutations identified in patients’ tumours and assess responsiveness to type I and II TKIs.

**Methods:**

We used structural modelling, in vitro kinase and in cell‐based assays to assess the response of MET mutations to type I and II TKIs. We then translated our pre‐clinical findings and treated patients with MET mutant tumours with selected inhibitors.

**Results:**

We detected the emergence of four (three previously uncharacterised and one known) MET resistance mutations (MET^G1090A^, MET^D1213H^, MET^R1227K^ and a MET^Y1230S^) in samples from patients with multiple solid tumours, including patients who had been previously treated with type I inhibitors.

In silico modelling and biochemical assays across a variety of MET alterations, including the uncharacterised MET^G1090A^ and the MET^Y1230S^ substitutions, demonstrated impaired binding of type I but not of type II TKIs (i.e., cabozantinib/foretinib). Applying our pre‐clinical findings, we then treated two patients (one with a non‐small‐cell lung cancer and one with a renal cell carcinoma) whose tumours harboured these previously uncharacterised MET alterations with cabozantinib, a type II MET TKI, and observed clinical responses.

**Conclusions:**

Comprehensive characterisation of the sensitivity of mutations to different TKI classes in oncogenic kinases may guide clinical intervention and overcome resistance to targeted therapies in selected cases.

**Key points:**

- Kinase mutations in RTKs are primary or secondary drivers in multiple cancer types
- Some of these mutations confer resistance to type I but not to type II inhibitors in preclinical samples and in patients
- The biochemical characterization of mutations in oncogenic kinases based on their sensitivity to type I and type II inhibitors is crucial to inform clinical intervention

## Graphical abstract

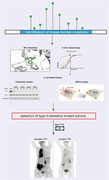

## 1 INTRODUCTION

MET alterations are known oncogenic drivers across a variety of tumours^1^, ^2^, ^3^, ^4^, ^5^, ^6^, ^7^ and can occur de novo or appear as acquired mechanisms of resistance to previous therapies. Gene amplifications, mutations and gene fusions can all lead to hyperactivation of MET,^4^ and may show distinct patterns of sensitivity and resistance to currently available MET‐directed therapies.

The incidence and biology of distinct MET alterations differ. De novo MET amplifications are identified in 1%–5% of non‐small‐cell lung cancers (NSCLCs), while acquired *MET* amplifications are found in up to 20% of patients with EGFR mutant NSCLC at progression to an anti‐EGFR tyrosine kinase inhibitor (TKI).^4^ In addition, MET amplifications have been reported in patients with ALK, ROS1, TRKA/B/C or RET fusion‐positive tumours at progression on TKI therapy.^8^

MET can also promote oncogenesis through the presence of activating mutations and/or mutations that generate splice variants of the MET transcript. The isoforms generated by the splice variants, specifically, lack exon 14 and are defined as MET exon 14 skipping variants.^9^, ^10^ Exon 14 contains the residue Y1003 which is the binding site for c‐CBL, the E3 ligase that promotes the ubiquitylation and degradation of the MET receptor. The absence of this residue thus interferes with the degradation of MET and results in sustained MET signalling.^11^ De novo MET exon 14 skipping variants are found in 3%–4% of NSCLC. These alterations can also emerge when patients with EGFR‐mutant NSCLC progress on EGFR TKIs.^12^

In addition, MET activation can also result from the presence of a MET fusion. MET fusions have been identified in several tumour types including gastric cancer, melanoma, thyroid carcinoma, papillary renal cell carcinoma (RCC), lung adenocarcinoma, hepatocellular carcinoma, glioma, colorectal cancer and sarcoma. Oncogenic MET fusions always include the intact kinase domain of MET and are constitutively active in a ligand‐independent manner.^13^, ^14^, ^15^, ^16^, ^17^

Several MET TKIs are currently used to treat MET altered tumours, with variable levels of activity shown for each compound against different MET alterations.^18^, ^19^, ^20^, ^21^, ^22^, ^23^ These TKIs include type I and type II agents. Type I MET TKIs are ATP competitors which bind to the active or DFG‐in conformation of the MET kinase domain.^24^, ^25^ These are divided into two categories based on their binding sites: type Ia (i.e., crizotinib) and type Ib (i.e., capmatinib, tepotinib and savolitinib) with the latter exhibiting higher specificity and thus representing the most commonly used in patients.^26^ Type II MET TKIs (i.e., cabozantinib, merestinib, foretinib and glesatinib) bind to the inactive or DFG‐out state of the MET kinase domain by extending to a hydrophobic back pocket.^24^, ^27^, ^28^

While each class of inhibitor has potential activity, resistance also represents a known liability.^18^, ^19^, ^20^, ^21^ Resistance can involve the bypass activation of compensatory pathways driven by KRAS, BRAF or EGFR^29^, ^30^ or can emerge via ‘on‐target’ mechanisms, whereby mutations develop in MET and compromise drug binding.^24^, ^31^, ^32^, ^33^

Acquired MET kinase domain mutations in residues MET^D1228^ and MET^Y1230^ confer resistance to type I MET TKIs in vitro by disrupting interactions between the drug and the MET kinase domain.^31^, ^32^ Importantly, examples exist that suggest that some MET mutations which confer resistance to type I MET TKIs remain sensitive to type II drugs and vice versa, thus suggesting that switching the class of inhibitor can overcome on‐target resistance in selected cases.^31^ The solvent front G1163R mutation, while conferring resistance to the type Ia inhibitor crizotinib, for example, remains sensitive to type Ib MET TKIs like tepotinib, savolitinib or capmatinib.^24^ By contrast, resistance to type II MET TKIs (i.e., glesatinib) can occur via mutations affecting residues L1195 and F1200.^24,^ ^27^

Here, we describe the identification of four MET resistance mutations which emerged clinically as mechanisms of secondary resistance to targeted therapy. We combined molecular dynamics (MD) simulations, in vitro kinase assays, and in‐cell assays to characterise these and other MET mutants for their responses to type I and type II MET inhibitors. Applying our pre‐clinical findings, we then treated patients with MET mutant tumours with selected MET TKIs.

## 2 MATERIALS AND METHODS

### 2.1 Patient samples and next generation sequencing

Patients at Memorial Sloan Kettering Cancer Center with alterations in MET were identified. Patients’ tumours were sequenced using the MSK‐IMPACT next generation sequencing platform, which at the time covered up to 468 genes of interest,^34^, ^35^ as well as with the RNA‐based ARCHER fusion panel.^36^, ^37^ Liquid biopsies were performed using our institution's MSK‐ACCESS assay, which covers 169 genes and accounts for paired leukocyte sequencing.^38^ Additional patients with MET alterations were identified from our clinical sequencing database, which amalgamates data on sequencing of tumours and/or ctDNA.^39^, ^40^ This study was approved by the Institutional Review Board of Memorial Sloan Kettering Cancer Center.

### 2.2 Compounds

Crizotinib (HY‐50878), capmatinib (HY‐13404), cabozantinib (HY‐13016) and foretinib (HY‐10338) were purchased from MedChemExpress.

### 2.3 Immunohistochemistry

Immunohistochemical staining for MET was carried out with the SP44 clone (Ventana/Roche), according to previously described methods.^41^, ^42^

### 2.4 In vitro kinase assays

Recombinant MET wild type and G1090A, Y1230S and Y1230H mutant kinase domains were synthesised by Signalchem (Signalchem Biotech Inc.). Kinases were tested against a titration of crizotinib, capmatinib and cabozantinib (with 2 µM being the highest concentration and 1:3 serial dilutions) in duplicate in the presence of 10 µM fixed ATP concentration by Reaction Biology (Reaction Biology Corporation). Results are presented as % activity (mean ± SD), with activity of 100% in the untreated kinases used as controls.

### 2.5 Plasmid generation, cell culture and Western blots

The CD47‐MET, the CD47‐MET G1090A and the CD47‐MET Y1230S‐encoding PUC‐GW‐Kan pDONR plasmids were generated by Genewiz. Gateway™ LR Clonase™ II Enzyme mix (Invitrogen; #11791020) was used to clone the above from pDONR into the plenti302 destination vector. These vectors were used to transiently transfect HEK‐293T cells or H3122 cells (ALK+ NSCLC) using Lipofectamine™ 3000 Transfection Reagent (Thermo Fisher; #L30000008) according to the manufacturer's protocol. Twenty‐four hours later transfected cells were treated with the indicated concentrations of the different MET inhibitors for 30 min. After incubation, cells were frozen. The day after incubation, protein lysates were extracted, quantified and used for Western blots. The following antibodies were used: Met (D1C2) XP® Rabbit mAb (CST, #8198S), Phospho‐Met (Tyr1234/1235) (D26) XP® Rabbit mAb (CST, #3077S), ERK (CST, #4695S), pERK T202/Y204 (CST, #9101S) and actin (CST, #4970S). Experiments shown are representative of three biological replicates. Western blots quantifications and analyses were performed using GraphPad 9.2.

### 2.6 Docking and molecular dynamic simulations

Available structures of MET kinase were used to construct a structural model of the kinase domain of MET (MET) in the active (PDB: 3Q6W,) and inactive forms (PDB: 6SD9). Structural models of MET–inhibitor complexes were generated using the co‐crystal structures of either the inhibitor (PDB: 2WGJ: Crizotinib; 5EOB: capmatinib, 5HTI: cabozantinib, 6SD9: foretinib, or of an analogue of the inhibitor (compounds closely related to the inhibitor) bound with MET kinase. Models of the mutant MET kinases in its apo form and in complex with inhibitors were generated using the corresponding wild type structures. The resulting models were further refined using MD simulations with the pmemd.cuda module of the program Amber20.^43^ The partial charges and force field parameters for each inhibitor were generated using the Antechamber module in Amber20. All atom versions of the Amber14SB force field (ff14SB)^44^ and the general Amber force field (GAFF)^45^ were used for the proteins and the inhibitors, respectively. MD simulations were carried out with a standard protocol.^46^ Simulation trajectories were visualised using VMD^47^ and PyMOL.^48^ Analyses were carried out using in‐house scripts and the ptraj module of Amber20. Relative binding free energies were calculated using the thermodynamic cycle, by calculating the differences in the free energy for the binding of an inhibitor to the wild type and to the mutant proteins.^49^

### 2.7 NanoBret target engagement intracellular kinase assay

NanoBret target engagement (TE) intracellular Kinase assay K10 adherent format was purchased from Promega (cat # N2521) as well as the Met Nanoluc construct (cat # NV1751). Met Nanoluc was mutagenised to generate the G1090A, Y1230H and Y1230S mutations using the Phusion site directed mutagenesis kit (Thermofisher cat #F541) following the manufacturer's instructions. HEK293T cells were then counted and resuspended at 2 × 10^5^ cells per mL, transfected with the above constructs with lipofectamine 3000 (Thermofisher cat #L3000015) and plated in polystyrene white plates (Corning cat # 3917) at 2 × 10^4^ cells per well. Past 30 h, a final concentration of  .25 uM of a cell‐permeable fluorescent tracer was added to the cells as well as serial dilutions of a Type I crizotinib or Type II cabozantinib (1 µM:2) test compounds. The cells were incubated at 37°C for 2 h. After equilibrating the plates at room temperature for 15 min, 50 uL of a solution containing complete substrate and inhibitor solution was added to the wells and incubated for 3 min. Donor NanoLuc signal (460 nm) and Acceptor fluorescent Tracer emission (618 nm) were read on a GloMax multi detection Plate Reader. MilliBRET ratio was calculated as follow: [Acceptor/Donor × 1000].

## 3 RESULTS

### 3.1 Identification of MET mutations in clinical samples

We identified the emergence of four (three previously uncharacterised and one known) MET mutations (MET^G1090A^, MET^D1213H^, MET^R1227K^ and a MET^Y1230S^) in samples from patients with multiple solid tumours, including patients who had been previously treated with MET inhibitors. Importantly, two of these mutations, the MET^G1090A^ and the MET^Y1230S^ substitutions, were found in more than 1 patient, indicating that they are recurrent events.

**FIGURE 1:**
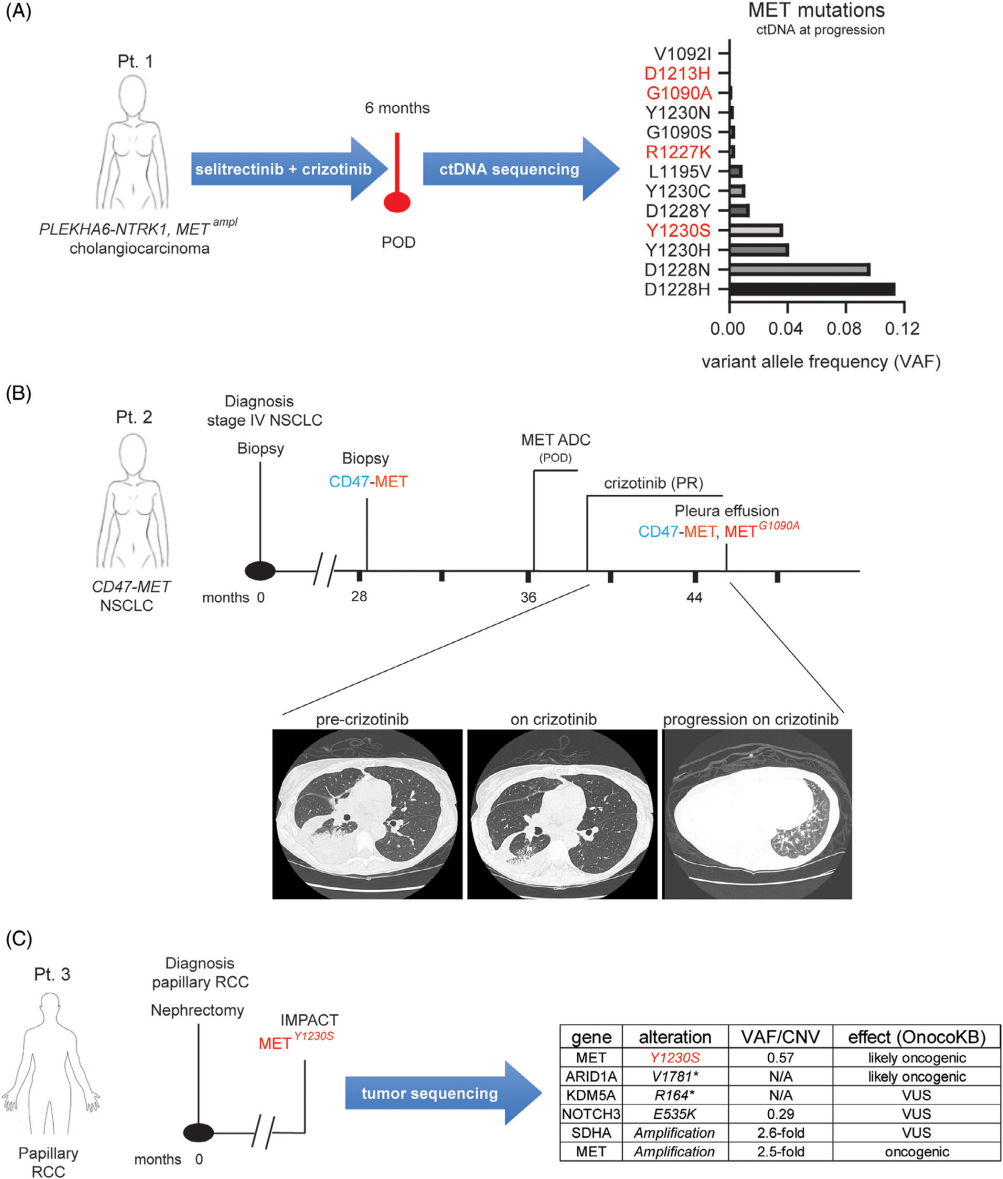
Identification of previously uncharacterised MET tyrosine kinase inhibitor (TKI)‐resistance mutations in patients. (A) Relevant clinical treatment history of a patient (Pt. 1) with a stage IV, *PLEKHA6‐NTRK1* fusion‐positive, *MET* amplified cholangiocarcinoma. Pt. 1 was treated with the combination of selitrectinib and crizotinib to which she responded. Six months later, she developed resistance. Sequencing of the ctDNA by MSK‐ACCESS at progression detected the presence of 13 new MET mutations, four of which (highlighted in red) revealed novel alterations including a MET^G1090A^, a MET^D1213H^, a MET^R1227K^ and a MET^Y1230S^ substitution. The variant allele frequency (VAF) for each alteration is indicated. (B) Clinical treatment history of a patient (Pt. 2) with stage IV, *CD47‐MET* fusion‐positive non‐small‐cell lung cancer (NSCLC). The timeline reports just those regimens that are relevant for this study. The emergence of the MET^G1090A^ found on a pleural effusion sample on crizotinib progression is indicated. Computed tomography (CT) scans pre‐crizotinib, on‐crizotinib and following crizotinib progression are presented. POD, progression of disease; PR, partial response. (C) Clinical history of a patient (Pt. 3) with a MET^Y1230S^ mutant renal cell carcinoma (RCC). Targeted sequencing results performed by MSK‐IMPACT on the tumour are listed.

In one patient (Pt. 1 in figures) all four mutations were detected in the ctDNA collected at progression to targeted therapy. Specifically, this patient had stage IV *PLEKHA6‐NTRK1*, *MET* amplified (fold change: 12.3) cholangiocarcinoma which initially responded and then became resistant to the combination therapy with the TRK inhibitor selitrectinib and the type I MET inhibitor crizotinib. As our group previously reported,^50^ ctDNA sequencing at progression (Table S1) uncovered the emergence of 13 different MET mutations, including some known to confer resistance to crizotinib (i.e., MET^D1228H^, MET^Y1230S^)^33^ and three that have only been previously described in this patient and which have yet to undergo extensive clinical and structural characterisation (i.e., MET^G1090A^, MET^D1213H^, MET^R1227K^; Figure 1A).

Patient 2 (Pt. 2) was a 61‐year‐old female, never smoker, who was initially diagnosed with stage IV NSCLC (Figure 1B). A pleural biopsy at the time showed only a TP53 mutation on DNA sequencing. She was initially treated on an immunotherapy trial, but had rapid progression within 2 months. She was then treated with carboplatin, pemetrexed and bevacizumab followed by pemetrexed/bevacizumab maintenance for just over 7 months, prior to stopping for renal insufficiency and being followed with expectant management. One year and 4 months later, she underwent an ablation for progression of disease in the lung. At the time, next generation sequencing of a right lung specimen showed a CD47‐MET rearrangement as well as a new MYH15‐MET rearrangement (Figure 1B and Table S2). Additional alterations were seen in TP53 and DOT1L, with APC and DNMT3A alterations in the matched normal blood (Table S2). The RNA‐based ARCHER fusion panel showed only the CD47‐MET rearrangement (Figure S1A). High MET expression was confirmed by immunohistochemistry (IHC) that showed both diffused cytoplasmic and membranous subcellular localisation of the fusion (Figure S1B). Approximately 8 months later, the patient required further systemic therapy and was started on a MET‐directed antibody–drug–conjugate (ADC) trial, but did not respond, likely due to the absence of the MET extracellular ADC binding site in the MET fused protein. She was then treated with crizotinib, to which she responded (Figure 1B). After 5 months on therapy, she developed a significant pleural effusion that required pleurX placement. Sequencing of the DNA collected from the fluid‐derived cell pellet showed a MET^G1090A^ mutation, in addition to the CD47‐MET rearrangement (Figures 1B and S1A). Other alterations are shown in Table S3. A plan was made to add chemotherapy to crizotinib given her clinically significant pleural effusion which was felt to be consistent with progression. However, in light of concerns about the risks of immune suppression due to chemotherapy during the Covid pandemic and her relative stability following pleurX placement, she was maintained on crizotinib alone. Nearly a year after the initial MET^G1090A^ was discovered, she developed near complete radiographic consolidation of her right lung.

In addition to patient 1, the MET^Y1230S^ mutation was identified via IMPACT sequencing in the tumour of a 53‐year‐old male patient (Pt. 3) with resected papillary RCC (Figure 1C). Additional relevant alterations identified included a frameshift in the epigenetic regulator ARID1A and MET copy gain (2.5‐fold; Figure 1C). The patient was enrolled in a trial testing the activity of the combination of everolimus and bevacizumab for non‐clear cell kidney cancers. After an initial mixed response (stable disease by RECIST), tumour resistance was observed about 200 days later and treatment was discontinued.

### 3.2 MET^G1090A^ and MET^Y1230S^ mutations confer resistance to type I but not type II MET inhibitors

**FIGURE 2:**
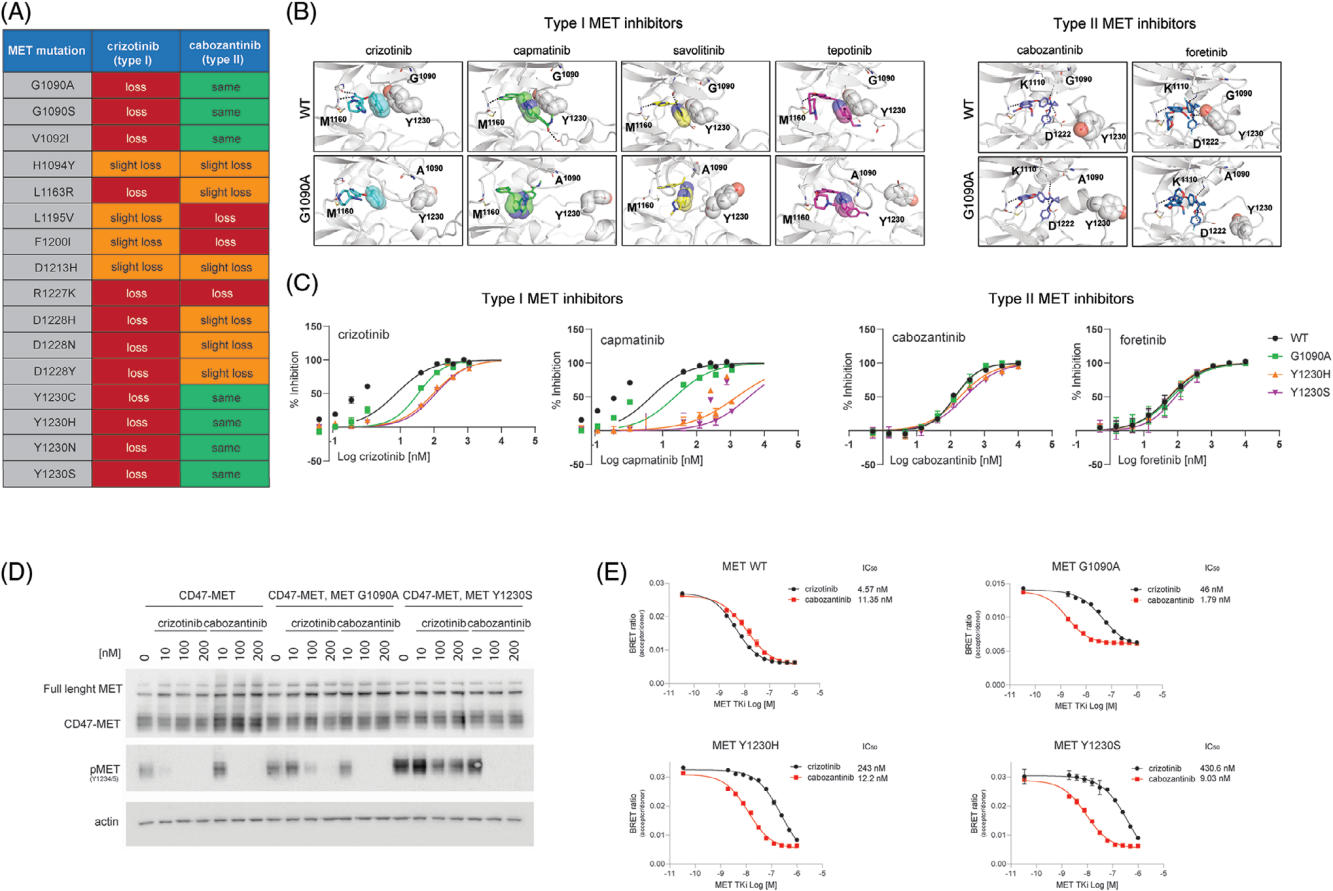
Sensitivity of MET resistance mutations to type I and type II MET inhibitors. (A) Computational prediction of change in binding free energy (ΔΔ*G*) of MET resistance mutations to representative type I (crizotinib) and type II (cabozantinib) MET inhibitors using FEP/MBAR.^43^ The change in free energy (ΔΔ*G*) is highlighted in different colours (Green: ΔΔ*G* = ±.6 kcal/mol; Orange: ΔΔ*G* = .6–2.0 kcal/mol; Red: ΔΔ*G* > 2.0 kcal/mol). (B) Representative models showing type I and type II MET inhibitors in complex with MET WT and MET G1090A. Bound inhibitors (colour sticks) and kinase residue Y1230 (sphere) are displayed. Chemical groups of inhibitors that clash with mutant MET kinase are depicted as spheres for visualisation purposes. (C) In vitro kinase assays showing the activity of the type I MET inhibitors crizotinib and capmatinib and the type II MET inhibitors cabozantinib and foretinib against recombinant WT and mutant (MET^G1090A^, MET^Y1230S^, MET^Y1230H^) MET kinases. (D) Western blot images depicting the activity of crizotinib or cabozantinib against HEK‐293T cells transfected with CD47‐MET, CD47‐MET, MET^G1090A^ and CD47‐MET, MET^Y1230S^‐encoding plasmids. (E) NanoBret analysis following transfection of HEK‐293T cells with CD47‐MET, CD47‐MET, MET^G1090A^, CD47‐MET, MET^Y1230S^ and CD47‐MET, MET^Y1230H^‐encoding plasmids and treatment with increasing concentrations of the type I MET tyrosine kinase inhibitor (TKI) crizotinib or the type II MET TKI cabozantinib.

Given that each patient had a potentially targetable MET alteration, we carried out pre‐clinical studies to identify whether MET‐directed therapy would be predicted to offer benefit and whether specific inhibitors could be used for these patients. Anecdotal examples have demonstrated that some MET mutations that confer resistance to type I MET inhibitors may still respond to type II agents (e.g., MET^D1228H^, MET^Y1230C^).^29^, ^31^ Hence, we performed in silico molecular modelling combined with MD simulations to predict the binding of all reported MET resistance mutations (including the four MET mutations we identified; Figure 2A) to a representative type I (crizotinib) and a representative type II (cabozantinib) MET inhibitor.

Notably, we identified additional opportunities for therapeutic intervention for patients with certain MET alterations. We found that some MET mutations, including MET^G1090A^ and MET^Y1230S^, are predicted to impair binding of type I but not type II MET inhibitors (Figure 2A). Our findings suggest that tumours with specific MET mutations may benefit from MET‐directed therapy that uses a type II agent.

Specifically, in the case of the MET^G1090A^ substitution, our simulations indicated that the 2,6‐dichloro‐3‐fluorophenyl moiety present on crizotinib and other type I MET inhibitors directly bind through a pi stacking interaction to the side chain of the Y1230 residue in the activation loop of the MET wild type kinase. In the MET^G1090A^ mutant kinase, the position of the Y1230 residue is predicted to be flipped by roughly 180°. As a result, the interaction between type I MET TKIs and the Y1230 is compromised; this, in turn, is predicted to reduce crizotinib's binding affinity for the mutant kinase (Figure 2B).

Conversely, the type II agents cabozantinib and foretinib do not interact with the Y1230 and therefore their binding affinity to the mutant MET kinase is predicted to be retained (Figure 2B). Similarly, in the case of the MET^Y1230S^, and as also previously shown for the MET^Y1230H^ mutation,^51^ the substitution of the Y1230 with a serine is predicted to disrupt the pi stacking interaction and thus weaken the binding of type I but not of type II MET inhibitors to the MET^Y1230S^ mutant kinase (Figure 2A). These findings suggested that the MET^G1090A^ and the MET^Y1230S^ mutant kinases may be resistant to type I inhibition, but not to type II MET inhibitors.

To further test our hypothesis, we performed in vitro kinase assays using a titration of crizotinib and capmatinib as representative type I agents and of cabozantinib and foretinib as representative type II drugs against MET WT and mutant MET^G1090A^, MET^Y1230S^ and MET^Y1230H^ (used as control) recombinant kinases (Figure 2C). IC_50_ values of 7.58 nM and 4.59 nM were obtained for the type I MET inhibitors crizotinib and capmatinib against MET WT. Significantly higher IC_50_ values were obtained when the same agents were tested against the MET^G1090A^ kinase (IC_50_ = 33.99 nM and 23.05 nM for crizotinib and capmatinib, respectively) or the MET^Y1230S^ kinase (IC_50_ = 112.6 nM and 3554 nM for crizotinib and capmatinib, respectively). Interestingly, while both type II MET inhibitors cabozantinib and foretinib were slightly less potent than type I agents against MET WT (IC_50_ = 133 nM and 57.05 nM for cabozantinib and foretinib, respectively), their activity was not significantly inhibited when they were tested against the MET^G1090A^ kinase (IC_50_ = 137 nM and 67.44 nM for cabozantinib and foretinib, respectively) or the MET^Y1230S^ kinase (IC_50_ = 268.1 nM and 86.13 nM for cabozantinib and foretinib, respectively; Figures 2C and 2SA).

We next transfected HEK‐293T cells with plasmids encoding the CD47‐MET fusion, the CD47‐MET fusion harbouring the G1090A mutation, or the CD47‐MET fusion harbouring the Y1230S mutation and evaluated changes in MET phosphorylation following treatment with type I and type II agents. Our results showed that, while crizotinib (type I) and cabozantinib (type II) were equally effective in inhibiting MET phosphorylation in cells transfected with the fusion alone, only type II inhibition with cabozantinib impeded MET activity at low nanomolar concentrations in cells transfected with the mutant MET fusions (Figures 2D and S2B,C). Similar confirmatory results were obtained when the type I capmatinib and the type II foretinib were tested against the same constructs (Figure S2D,E). These in‐cell data further validated that type II MET inhibitors retain activity against the MET^G1090A^ and the MET^Y1230S^ mutant kinases.

While our in‐cell data using the HEK‐293T cells suggest that the differential response to type I and type II MET inhibitors depends on the specific MET substitution, we proceeded to assess the possibility that tissue context in which the mutants are identified or the presence of co‐occurring drivers could influence drug sensitivity. Thus, we transfected the ALK fusion‐positive NSCLC cell line H3122 with the MET^G1090A^ and the MET^Y1230S^ mutations and confirmed these ALK fusion‐positive, MET mutant cell lines to be resistant to type I but not to type II MET, mimicking results we obtained in the HEK‐293T cell line (Figure S3).

We then performed NanoBret experiments to evaluate the binding affinity of type I and type II inhibitors for WT and mutant MET kinases. This technique allows derivation of binding affinities of drugs through *in‐cell* experiments following transfection with WT and mutant MET constructs and treatment with increasing concentrations of drugs. Data obtained in HEK‐293T cells show that cabozantinib binds MET G1090A, MET Y1230S and MET Y1230H kinases with higher affinity than crizotinib (Figure 2E). Similar results were obtained when the same NanoBret assay was conducted on the same cell model using a titration of the type I MET TKI capmatinib and the type II MET TKI cabozantinib (Figure S4).

### 3.3 MET^D1213H^ and MET^R1227K^ mutations do not impact the activity of type I or type II MET inhibitors

**FIGURE 3:**
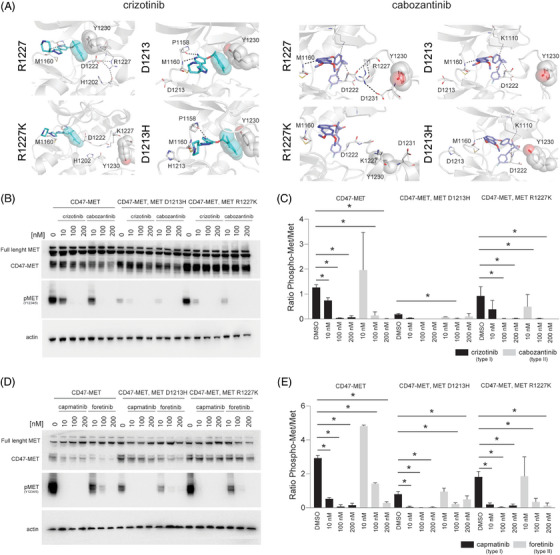
(A) Representative models showing type I and type II MET inhibitors in complex with MET WT, MET R1227K and MET D1213H kinases. Bound inhibitors (colour sticks) and relevant kinase residues (sphere) are displayed. (B–E) Western blot images (B, D) and their quantifications (C, E) depicting the activity of crizotinib or cabozantinib (B, C) or capmatinib and foretinib (D, E) against HEK‐293T cells transfected with CD47‐MET, CD47‐MET, MET^R1227K^ and CD47‐MET, MET^D1213H^‐encoding plasmids.

A different scenario was observed when we modelled the previously uncharacterised MET^R1227K^ and MET^D1213H^ mutations. Our predictions showed that these mutations would just slightly impact type I and type II TKIs response (Figure 3A). Specifically, for the MET^R1227K^ we found that Arg1227 is involved in a network of interactions with residues Asp1222, Phe1223, Gly1224, His1202 that partially stabilises the bound conformation of both crizotinib and cabozantinib. Replacement of Arg1227 with Lys may partially, but just slightly perturb this network of interactions. Similarly, the MET^D1213H^ mutation does not seem to destabilise the bound conformations of either crizotinib or cabozantinib; however, the interaction of crizotinib with Met1160 is not retained in the case of MET^D1213H^, suggesting this mutation to slightly impair crizotinib binding (Figure 3A). To evaluate the effect that these mutations have on the response to type I and type II MET inhibitors in cells, we transfected HEK‐293T cells with plasmids encoding the CD47‐MET fusion, the CD47‐MET fusion harbouring the R1227K mutation, or the CD47‐MET fusion harbouring the D1213H mutation and evaluated changes in MET phosphorylation following treatment with type I and type II agents. Consistent with our predictions, the presence of each of these mutations did not affect the ability of either type I (i.e., crizotinib, capmatinib) or type II (i.e., cabozantinib, foretinib) MET inhibitors to inhibit MET phosphorylation (Figure 3B–E).

Together, these data indicate that MET^R1227K^ and MET^D1213H^ mutations do not confer significant resistance to any of the type I or the type II MET TKIs tested and suggest that the presence of these MET mutations in the ctDNA of patient 1 (see Figure 1A) was not the main aetiology of resistance to crizotinib.

### 3.4 Cabozantinib is active against selected type I agents‐resistant MET mutants in patients

Given our pre‐clinical findings suggesting that some patients may continue to benefit from MET‐directed therapy even after developing alterations in MET known to confer resistance to certain TKIs, we aimed to identify patients who might benefit from treatment with a type II MET inhibitor.

**FIGURE 4:**
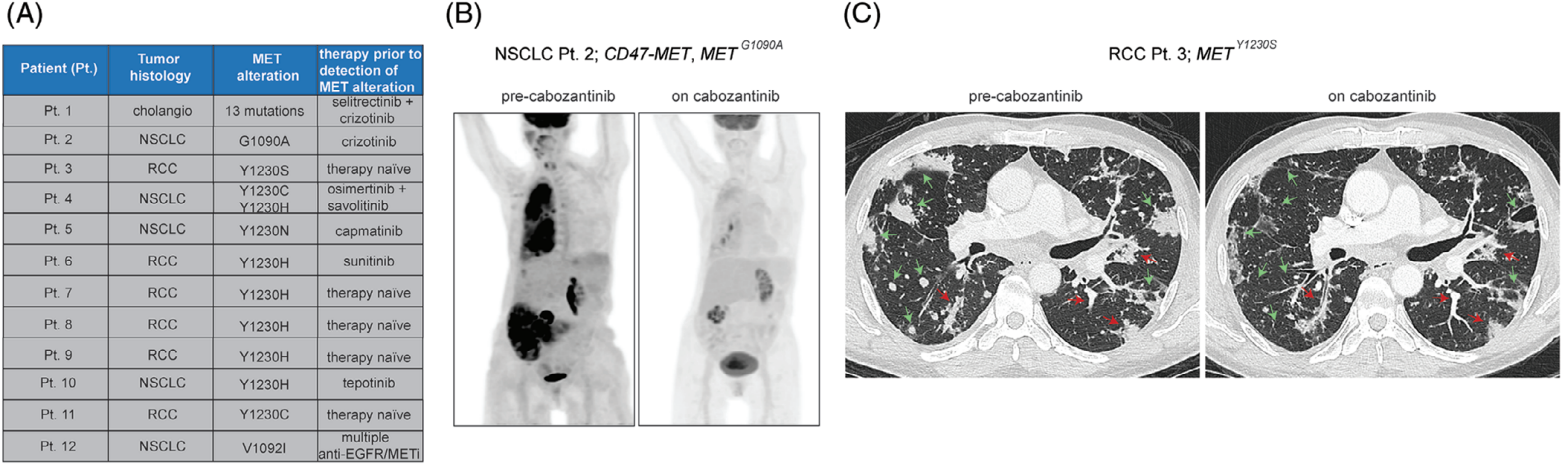
(A) List of patients identified whose tumours harbour MET mutations predicted to be sensitive to type II MET inhibitors. (B) PET scans showing the response of Pt. 2 (CD47‐MET, MET^G1090A^ mutant non‐small‐cell lung cancer [NSCLC]) to cabozantinib. (C) Computed tomography (CT) scans showing the response of Pt. 3 (MET^Y1230S^ mutant renal cell carcinoma [RCC]) to cabozantinib.

We surveyed our clinical database at MSKCC for patients with MET mutations predicted by our MD simulations and/or cell‐line data to respond to type II MET inhibitors. In addition to our three index patients (Pts. 1, 2 and 3), we found nine additional patients: five with RCC and four with NSCLC (Figure 4A). All patients with NSCLC with these alterations in their tumours had progressed on at least one line of therapy, suggesting that the MET mutation was the likely cause of the acquired resistance to prior therapy. By contrast, most of the RCC patients were treatment‐naïve (Figure 4A).

Two of the patients (Pts. 2 and 3) were considered candidates for additional therapy. Pt. 2, who had the CD47‐MET fusion‐positive, MET G1090A mutant NSCLC, and Pt. 3 with the MET Y1230S RCC, both clinically responded to the type II inhibitor cabozantinib (Figure 4B,C).

Unfortunately, despite the robust anti‐neoplastic response experienced by Pt. 2, she was unable to tolerate the medication at prescribed doses due to hand–foot syndrome, which eventually led to every‐other‐day dosing and then treatment discontinuation. Pt. 3 also developed skin toxicity and underwent dose reduction. While tolerability issues limited the time on treatment with cabozantinib, together these data provide clinical proof of principle suggesting that treating patients with type II MET TKIs can be beneficial in molecularly selected cases and emphasise the importance of developing tolerable type II inhibitors.

## 4 DISCUSSION

A variety of alterations in MET have been reported as oncogenic drivers, including amplifications, mutations and fusions.^1^, ^3^, ^4^, ^6^, ^52^ Resistance to targeted therapies for tumours with MET exon 14 skipping mutations or MET amplifications have been described, including bypass track alterations in both the MAPK and PI3K pathways, as well as the emergence of solvent front mutations and other alterations impacting the binding of type I inhibitors.^6^, ^24^, ^31^, ^32^, ^53^, ^54^, ^55^, ^56^, ^57^, ^58^, ^59^ Here, we report the identification and the biochemical characterisation of four MET mutations (a MET^G1090A^, a MET^D1213H^, a MET^R1227K^ and a MET^Y1230S^ substitution). Importantly, three of these MET mutations (MET^G1090A^, a MET^D1213H^, a MET^R1227K^) have never been structurally characterised in patients to date. We demonstrated that the use of type II MET inhibitors can effectively target tumours with selected MET mutations.

These mutations were identified in a number of different tumour types suggesting that our findings may have potential implications for treatment of MET‐driven cancers in a histology agnostic manner. For example, all four of the mutations were detected in the ctDNA of a patient with an *NTRK* fusion‐positive, *MET* amplified cholangiocarcinoma that progressed on the combination of selitrectinib (TRKi) and crizotinib (METi). The MET^G1090A^ mutation was identified by next‐generation sequencing in tumour biopsies collected from a patient with a MET fusion‐positive NSCLC that was progressing on crizotinib, while the MET^Y1230S^ substitution was found in a treatment naïve RCC.

Our molecular modelling and in vitro assays suggested that both the MET^G1090A^ and the MET^Y1230S^, but not the MET^D1213H^ and the MET^R1227K^ substitutions, would confer resistance to type I MET inhibitors. Specifically, our MD simulations revealed that the binding of type I MET inhibitors (i.e., crizotinib, capmatinib, savolitinib and tepotinib) to WT MET is stabilised by a pi stacking interaction between MET type I TKIs and the MET Y1230 residue. When the MET G1090, located in the P‐loop of MET, is mutated to an alanine, this substitution induces conformational changes that displace the MET Y1230 from its canonical position thus destabilising the interactions between MET type I TKIs and the MET^G1090A^ mutant kinase.

In keeping with prior work from our group and others on TKI type switching in fusion‐positive tumours,^31^, ^60^, ^61^, ^62^, ^63^, ^64^, ^65^ we also showed that the MET^G1090A^ mutation, while conferring resistance to type I MET inhibitors, does not affect the binding properties of type II TKIs. In other words, tumours with this mutation are resistant to agents that bind to the ATP pocket in the DFG‐in, active conformation of the MET kinase, but not to those drugs that bind to both the ATP pocket and the hydrophobic back pocket of the MET kinase in the DFG‐out (inactive) conformation. Consistently, the MET^G1090A^ mutant still responded to type II MET TKIs.

Similarly, our pre‐clinical data showed that the MET^Y1230S^ substitution disrupts the pi stacking interaction between type I TKIs and the MET kinase thus inducing type I but not type II MET TKIs resistance, as has been previously shown for the MET^Y1230H^ alteration. In agreement with our in vitro studies, when we treated Pt. 2 with the CD47‐MET, MET^G1090A^ mutant NSCLC and Pt. 3 with the MET^Y1230S^ RCC with the type II MET TKI cabozantinib we observed brisk and robust clinical responses. Importantly, while the MET^Y1230S^ mutation has been previously identified in patients and pre‐clinically characterised,^24^, ^33^ this is the first time that the clinical activity of a type II MET inhibitor against a tumour harbouring this MET alteration is reported.

**FIGURE 5:**
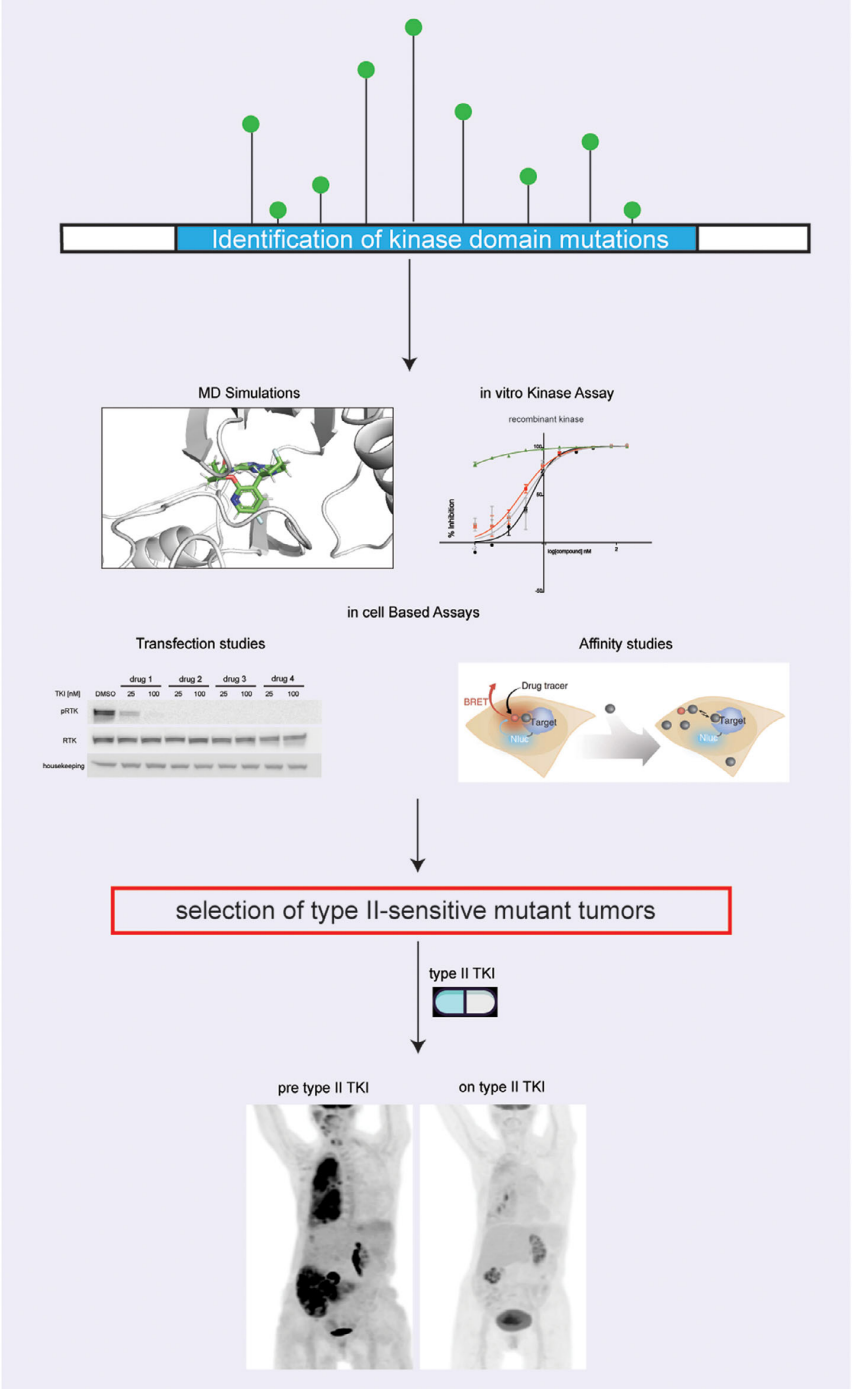
Graphic summarising steps taken to conduct this study: from the initial identification of MET mutations in clinical samples, to their pre‐clinical and biochemical characterisation, to the final design of ad hoc treatments for patients based on our laboratory findings.

We also characterised the sensitivity to type I and type II MET TKIs of all clinically reported resistance alterations in MET that we could find published to date. We found additional MET mutations which are predicted to confer resistance to type I drugs but that can still be managed by using a type II TKI (e.g., MET^G1090S^ and MET^V1092I^; Figure 2A). These results are consistent with previous recent studies^24^, ^32^, ^33^ and support the concept that an in‐depth biochemical characterisation of mutations in oncogenic kinases may enable tailoring of therapy for patients with both de novo and acquired alterations in MET and other oncogenes (Figure 5). Given the dearth of treatments for MET‐driven tumours, such type switching addresses an important clinical need by opening up new therapeutic options for patients with resistance alterations.

It is important to recognise, however, that both patients that we treated with cabozantinib developed significant side effects which resulted in initial drug dose reduction followed by treatment discontinuation. Cabozantinib is a type II multikinase inhibitor known to target MET and also other kinases. It is currently approved for the treatment of RCC because of its antiangiogenic properties.^66^ Previous clinical studies have shown that cabozantinib toxicity, which mainly manifests as diarrhoea, nausea, fatigue, decreased appetite, hand–foot syndrome and hypertension is due to the direct inhibition of VEGFR, FLT3 and KIT kinases, primary cabozantinib targets.^67^ This evidence, together with the fact that some resistance mutations in other cabozantinib secondary targets such as ALK, ROS1 and TRKA/B/C have also been shown to induce resistance to type I but not to type II TKIs^29^, ^31^, ^33^, ^60^, ^68^, ^69^, ^70^, ^71^ provide a strong rationale for the optimisation of more tolerable cabozantinib‐derived type II agents.

Our study is limited in that it includes data on MET resistance alterations from a single centre. Nonetheless, this study used a large, robust sequencing cohort to identify previously uncharacterised alterations in MET using a combination of MD simulation, cell‐line data and clinical data.

Overall, our results provide evidence of TKI type switching as a way to surmount resistance in patients with MET‐driven tumours and demonstrate the sensitivity of the MET^G1090A^, the MET^Y1230S^ as well as of other recurrent MET mutations to type II inhibition.

## AUTHOR CONTRIBUTIONS

**Yonina R. Murciano‐Goroff, Valentina Foglizzo, Srinivasaraghavan Kannan, Chandra S. Verma, Alexander Drilon and Emiliano Cocco**: Designed the study. **Yonina R. Murciano‐Goroff, Jason Chang, Natasha Rekhtman, Ann Elizabeth Sisk, Jamie Gibson, Lia Judka, Kristen Clemens, Estelamari Rodriguez, Madeline Merrill, Erica Sgroe, Matteo Repetto, Helena A. Yu, Zsofia K. Stadler and Michael F. Berger**: Helped with patients’ data analysis. **Valentina Foglizzo, Paola Roa, Shaza Sayed Ahmed, Nicole V. Bremer, Courtney Lynn Binaco, Sherifah Kemigisha Muzungu, Eneda Toska, Srinivasaraghavan Kannan**: Performed the experiments. **Srinivasaraghavan Kannan, Chandra S. Verma**: Performed the computational modelling. **Yonina R. Murciano‐Goroff, Valentina Foglizzo, Srinivasaraghavan Kannan, Chandra S. Verma, Alexander Drilon and Emiliano Cocco**: Drafted the manuscript.

## CONFLICT OF INTEREST STATEMENT

Yonina R. Murciano‐Goroff reports travel, accommodation and expenses from AstraZeneca and Loxo Oncology/Eli Lilly. She acknowledges honoraria from Virology Education and Projects in Knowledge (for a CME program funded by an educational grant from Amgen). She has been on an advisory board for Revolution Medicines, and consulted for AbbVie. She acknowledges associated research funding to the institution from Mirati Therapeutics, Bristol Myers Squibb/E.R. Squibb & Sons, Loxo Oncology at Eli Lilly, Elucida Oncology, Taiho Oncology, Hengrui USA, Ltd/Jiangsu Hengrui Pharmaceuticals, Luzsana Biotechnology, Endeavor Biomedicines and AbbVie. She is an employee of Memorial Sloan Kettering Cancer Center, which has an institutional interest in Elucida. She acknowledges royalties from Rutgers University Press and Wolters Kluwer. She acknowledges food/beverages from Endeavor Biomedicines, and other services from Amgen, AbbVie and Loxo Oncology/Eli Lilly. Yonina R. Murciano‐Goroff acknowledges receipt of training through an institutional K30 grant from the NIH (CTSA UL1TR00457). She has received funding from a Kristina M. Day Young Investigator Award from Conquer Cancer, the ASCO Foundation, endowed by Dr. Charles M. Baum and Carol A. Baum. She is also funded by the Fiona and Stanley Druckenmiller Center for Lung Cancer Research, the Andrew Sabin Family Foundation, the Society for MSK, the Squeri Grant for Drug Development and a Paul Calabresi Career Development Award for Clinical Oncology (NIH/NCI K12 CA184746) as well as through NIH/NCI R01 CA279264. Zsofia K. Stadler has intellectual property rights in SOPHiA Genetics and serves as an Associate Editor for JCO Precision Oncology and as a Section Editor for UpToDate. Zsofia K. Stadler's immediate family member serves on the Board of Directors for Adverum Biotechnologies, is Co‐Founder, CMO and President for Blue Gen Therapeutics Foundation, and serves as a consultant in Ophthalmology for Apellis, Novartis, Outlook Therapeutics, Optos and Regeneron outside the submitted work. Michael F. Berger acknowledges personal fees (AstraZeneca, Paige.AI), Research Support (Boundless Bio), Intellectual Property Rights (SOPHiA Genetics). Eneda Toska has grants from AstraZeneca and consulting fees from Menarini. Srinivasaraghavan Kannan and Chandra S. Verma are founder directors of SiNOPSEE Therapeutics and Aplomex. Alexander Drilon declares: HONORARIA: 14ner/Elevation Oncology, Amgen, Abbvie, AnHeart Therapeutics, ArcherDX, AstraZeneca, Beigene, BergenBio, Blueprint Medicines, Bristol Myers Squibb, Boehringer Ingelheim, Chugai Pharmaceutical, EcoR1, EMD Serono, Entos, Exelixis, Helsinn, Hengrui Therapeutics, Ignyta/Genentech/Roche, Janssen, Loxo/Bayer/Lilly, Merus, Monopteros, MonteRosa, Novartis, Nuvalent, Pfizer, Prelude, Regeneron, Repare RX, Springer Healthcare, Takeda/Ariad/Millennium, Treeline Bio, TP Therapeutics, Tyra Biosciences, Verastem, Zymeworks; ADVISORY BOARDS: Bayer, MonteRosa, Abbvie, EcoR1 Capital, LLC, Amgen, Helsinn, Novartis, Loxo/ Lilly, AnHeart Therapeutics, Nuvalent; CONSULTING: MonteRosa, Innocare, Boundless Bio, Treeline Bio, Nuvalent, 14ner/Elevation Oncology, Entos, Prelude; COPYRIGHT: Selpercatinib‐Osimertinib (filed/pending); EQUITY: mBrace, Treeline; ASSOCIATED RESEARCH PAID TO INSTITUTION: Foundation Medicine, GlaxoSmithKline, Teva, Taiho, PharmaMar; OTHER: Merck, Puma, Merus, Boehringer Ingelheim; ROYALTIES: Wolters Kluwer, UpToDate; CME HONORARIA: Answers in CME, Applied Pharmaceutical Science, Inc, AXIS, Clinical Care Options, Doc Congress, EPG Health, Harborside Nexus, I3 Health, Imedex, Liberum, Medendi, Medscape, Med Learning, MedTalks, MJH Life Sciences, MORE Health, Ology, OncLive, Paradigm, Peerview Institute, PeerVoice, Physicians Education, Projects in Knowledge, Resources, Remedica Ltd, Research to Practice, RV More, Targeted Oncology, TouchIME, WebMD; Emiliano Cocco declares: RESEARCH FUNDS: InnoCare Pharma, ERASCA and Prelude. Emiliano Cocco is also a consultant for ENTOS, Inc.

### ETHICS STATEMENT

This study was approved by the Institutional Review Board of the Memorial Sloan Kettering Cancer Center.

## Supporting information



Supporting Information

Supporting Information

Supporting Information

Supporting Information

Supporting Information

Supporting Information

Supporting Information

Supporting Information

## Data Availability

The data that support the findings of this study are available on request from the corresponding author. The data are not publicly available due to privacy or ethical restrictions.
