# Supplementary figures and images for: Responsiveness of different MET tumour alterations to type I and type II MET inhibitors
Source: Clin Transl Med. 2025 May 29;15(5):e70338. doi: 10.1002/ctm2.70338 (PMC12120261; doi:10.1002/ctm2.70338)

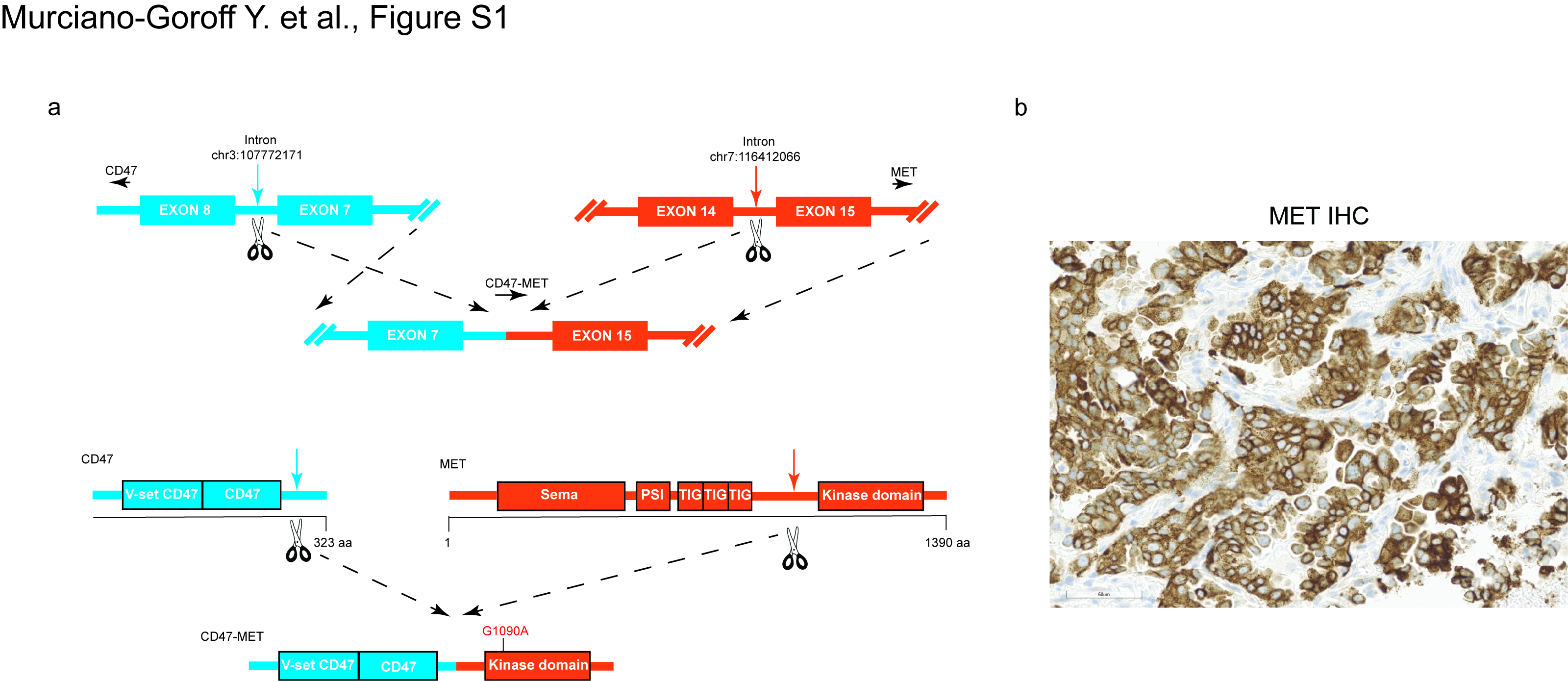

Supplement: Supplementary file 1 — Supporting Information [file CTM2-15-e70338-s003.tif]

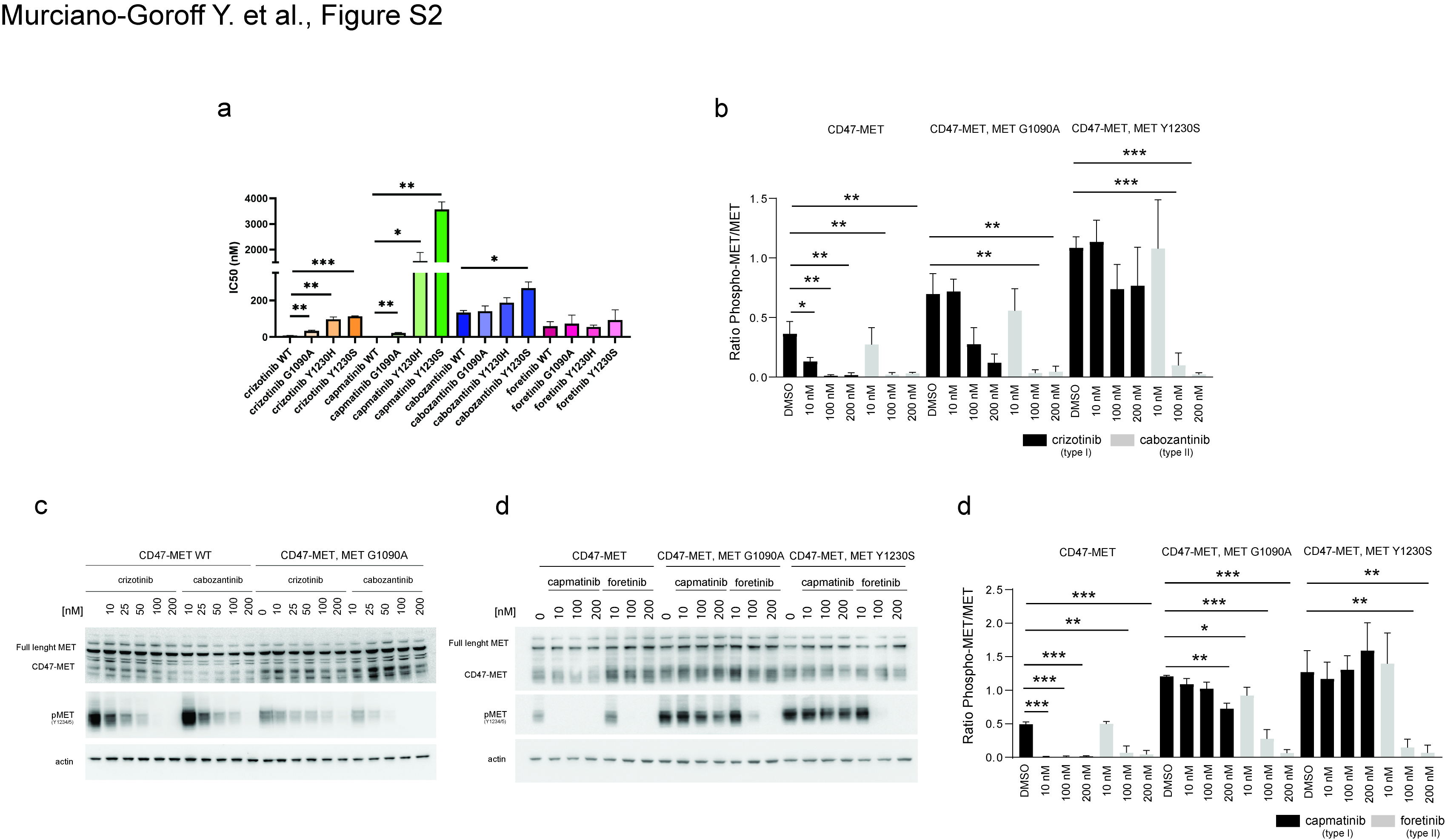

Supplement: Supplementary file 2 — Supporting Information [file CTM2-15-e70338-s006.tif]

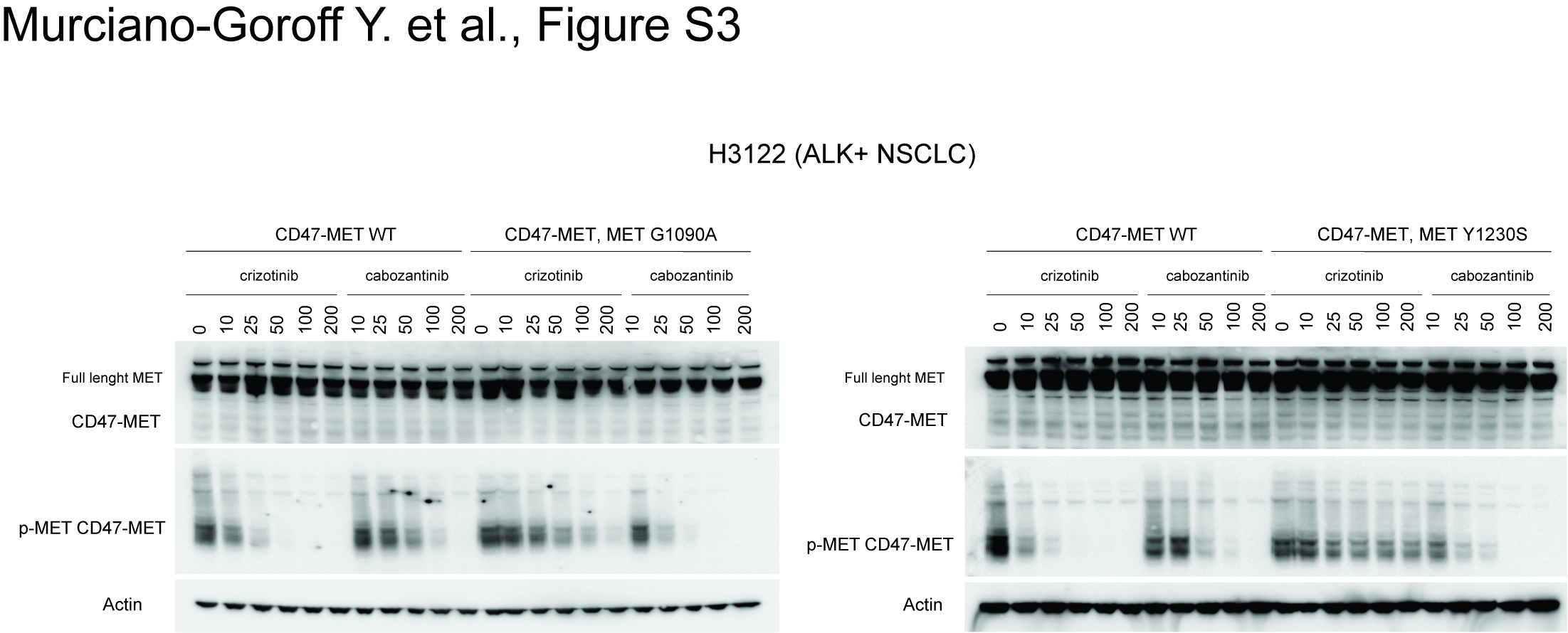

Supplement: Supplementary file 3 — Supporting Information [file CTM2-15-e70338-s008.tif]

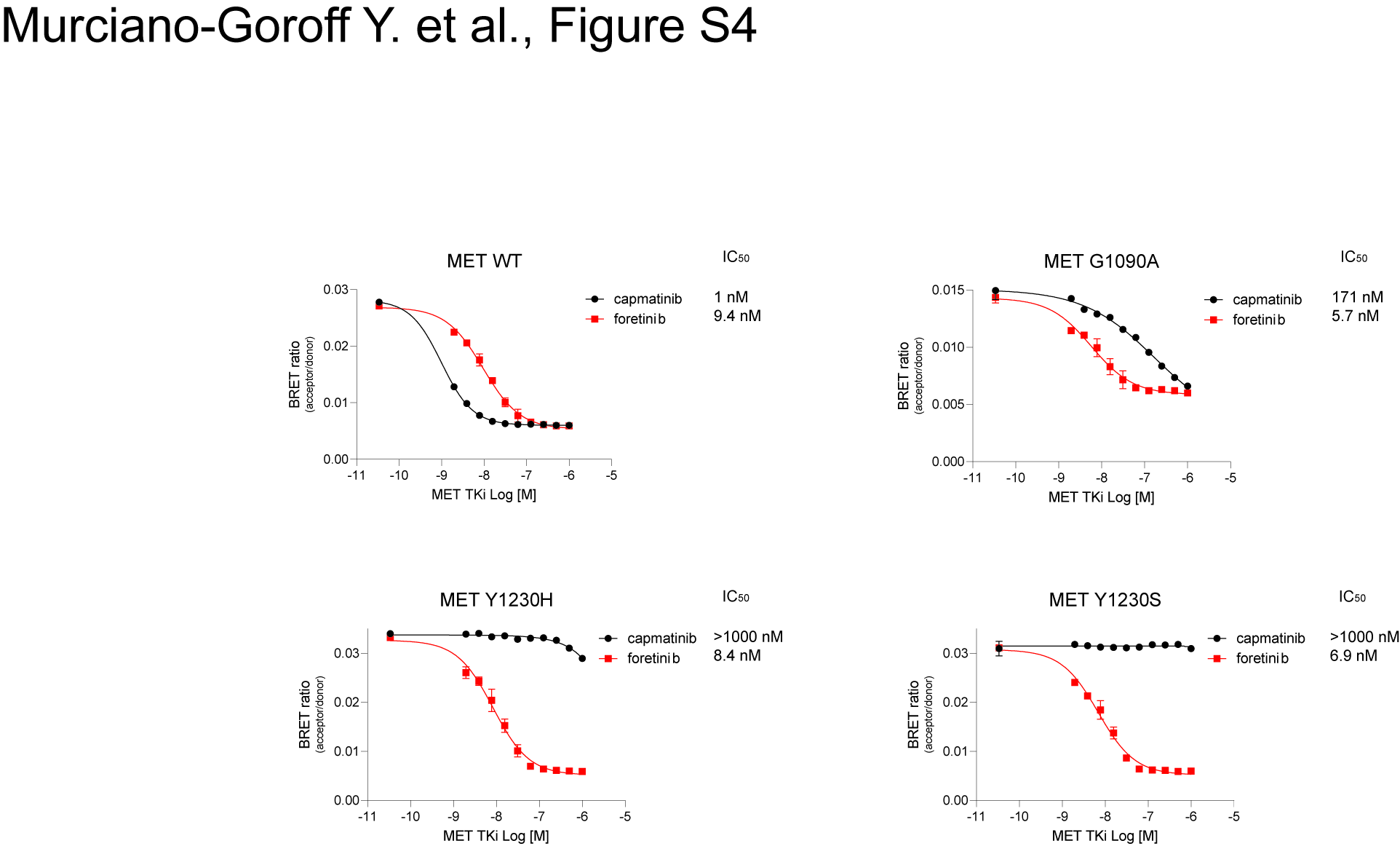

Supplement: Supplementary file 4 — Supporting Information [file CTM2-15-e70338-s001.tif]
