# Supplementary material for: Responsiveness of different MET tumour alterations to type I and type II MET inhibitors
Source: Clin Transl Med. 2025 May 29;15(5):e70338. doi: 10.1002/ctm2.70338 (PMC12120261; doi:10.1002/ctm2.70338)
Supplement: Supplementary file 5 — Supporting Information [file CTM2-15-e70338-s002.docx]

**Table S-1**. List of complete alterations detected in the ctDNA samples serially collected from Patient 1 (*NTRK* fusion-positive cholangiocarcinoma).

| *Alteration* | *Baseline on selitrectinib* | *POD on selitrectinib* | *On selitrectinib + crizotinib* | *POD on selitrectinib + crizotinib* |
| --- | --- | --- | --- | --- |
| NF1 S421F | 0.074 | 0.113 | 0 | 0.143 |
| EGFR S123F | 0.007 | 0.002 | 0 | 0.011 |
| MET D1228D | 0 | 0 | 0 | 0.097 |
| MET Y1230N | 0 | 0 | 0 | 0.003 |
| MET R1227K | 0 | 0 | 0 | 0.004 |
| MET Y1230C | 0 | 0 | 0 | 0.011 |
| MET D1228H | 0 | 0 | 0 | 0.114 |
| MET Y1230H | 0 | 0 | 0 | 0.041 |
| MET Y1230S | 0 | 0 | 0 | 0.037 |
| MET D1228Y | 0 | 0 | 0 | 0.014 |
| MET L1195V | 0 | 0 | 0 | 0.009 |
| MET G1090S | 0 | 0 | 0 | 0.004 |
| MET G1090A | 0 | 0 | 0 | 0.002 |
| MET D1213H | 0 | 0 | 0 | 0.001 |
| MET V1092I | 0 | 0 | 0 | 0.0002 |
