# Supplementary material for: Responsiveness of different MET tumour alterations to type I and type II MET inhibitors
Source: Clin Transl Med. 2025 May 29;15(5):e70338. doi: 10.1002/ctm2.70338 (PMC12120261; doi:10.1002/ctm2.70338)
Supplement: Supplementary file 6 — Supporting Information [file CTM2-15-e70338-s004.docx]

**Table S-2**. List of complete alterations detected in the pre-crizotinib NSCLC of Patient 2.

| gene | alteration |
| --- | --- |
| *CD47-MET* | rearrangement |
| *MYH15-MET* | rearrangement |
| *TP53* | H214L |
| *TP53* | R202C |
| *DOT1L* | rearrangement |
| *DNMT3A* | P256Gfs*5 (germline) |
| *DNMT3A* | C559Y (germline) |
| *APC* | N1533D (germline) |
