# Supplementary material for: Responsiveness of different MET tumour alterations to type I and type II MET inhibitors
Source: Clin Transl Med. 2025 May 29;15(5):e70338. doi: 10.1002/ctm2.70338 (PMC12120261; doi:10.1002/ctm2.70338)
Supplement: Supplementary file 7 — Supporting Information [file CTM2-15-e70338-s005.docx]

**Table S-3**. List of complete alterations detected in the post-crizotinib NSCLC of Patient 2.

| Gene | Alteration |
| --- | --- |
| *MET* | G1090A |
| *CD47-MET* | rearrangement |
| *MYH15-MET* | rearrangement (ARCHER negative) |
| *TP53* | H214L |
| *TP53* | R202C |
| *TERT* | AMP |
| *SDHA* | AMP |
| *TRIP13* | AMP |
| *CD79B* | AMP |
| *RPTOR* | AMP |
