# Supplementary material for: Responsiveness of different MET tumour alterations to type I and type II MET inhibitors
Source: Clin Transl Med. 2025 May 29;15(5):e70338. doi: 10.1002/ctm2.70338 (PMC12120261; doi:10.1002/ctm2.70338)
Supplement: Supplementary file 8 — Supporting Information [file CTM2-15-e70338-s007.docx]

**Table S-4**. List of complete alterations detected in the pre-cabozantinib RCC of Patient 3.

| Gene | Alteration |
| --- | --- |
| *MET* | Y1230S |
| *MET* | AMP |
| *SDHA* | AMP |
| *ARID1A* | V1781* |
| *KDM5A* | R164* |
| *NOTCH3* | E535K |
